# An initial melanoma diagnosis may increase the subsequent risk of prostate cancer: Results from the New South Wales Cancer Registry

**DOI:** 10.1038/s41598-018-25408-6

**Published:** 2018-05-08

**Authors:** D. Cole-Clark, V. Nair-Shalliker, A. Bang, K. Rasiah, V. Chalasani, D. P. Smith

**Affiliations:** 1 Department of Surgery, Royal North Shore Hospital, New South Wales, Australia; 2 Cancer Research Division, Cancer Council NSW, Sydney, NSW Australia; 3 Sydney School of Public Health, Sydney Medical School, The University of Sydney, Sydney, NSW Australia; 4 Northern Sydney Local Health District, New South Wales, Australia; 5 Garvan Institute of Medical Research & Kinghorn Cancer Centre, New South Wales, Australia; 6 Australian and New Zealand Urogenital and Prostate (ANZUP) Cancer Trials Group, Discipline of Surgery, University of Sydney, New South Wales, Australia; 7 Menzies Health Institute, Queensland, Griffith University, Gold Coast, Queensland Australia

## Abstract

Emerging evidence suggests that a diagnosis of cutaneous melanoma (CM) may be associated with prostate cancer (PC) incidence. We examined if the incidence of CM was associated with an increased subsequent risk of PC. We used data from the New South Wales Cancer Registry for all CM and PC cases diagnosed between January 1972 and December 2008. We calculated the age standardized incidence ratio (SIR) and 95% confidence intervals (95% CI) for PC incidence following a CM diagnosis, applying age- and calendar- specific rates to the appropriate person years at risk. We determined rate ratio (RR) and 95% CI of PC incidence according to specified socio-demographic categories and disease related characteristics, using a negative binomial model. There were 143,594 men diagnosed with PC or CM in the study period and of these 101,198 and 42,396 were diagnosed with PC and CM, respectively, as first primary cancers. Risk of PC incidence increased following CM diagnosis (n = 2,114; SIR = 1.25; 95% CI:1.20.8-1.31: p < 0.0001), with the increased risk apparent in men diagnosed with localised CM (n = 1,862;SIR = 1.26; 95% CI:1.20–1.32). CM diagnosis increased the subsequent risk of PC incidence. This raises the potential for future PC risk to be discussed with newly diagnosed males with CM.

## Introduction

Australia has the highest incidence rates of both prostate cancer (PC) and cutaneous melanoma (CM), internationally^1^. Prostate cancer is the most commonly registered cancer in Australian men with an estimated 16,655 new diagnoses in 2017^2^. Melanoma is the third most commonly diagnosed cancer in Australian men with approximately 8,392 new cases diagnosed in 2017^3^.

The few established risk factors for prostate cancer are generally considered non-modifiable such as advancing age, family history and African ancestry, with obesity being classified as an established risk factor for aggressive disease^4,5^. Several ecological studies have suggested, albeit with some uncertainty, that solar UV exposure maybe a potential risk factor of prostate cancer^6,7^, where studies from regions of low ambient UV showed a reduced risk of developing prostate cancer^8^, while those from regions of high ambient UV showed an increased risk^9,10^.

Sun exposure is the leading environmental cause of melanoma^11^. An estimated 65% of melanomas, and almost all keratinocytic cancers, in Australians, are attributable to high ambient UV levels, with attributable risks higher in men (69.9%) than in women (54.3%)^12,13^. Melanoma may also be associated with PC risk^9,10,14^. Using melanoma as a proxy for high sun exposure in a study of seven cancer registries, Tuohimaa showed that melanoma cases were at an increased risk of developing prostate cancer (SIR = 1.27; 95% CI: 1.20–1.33)^14^. Similar associations were also observed more recently in the Norwegian population (SIR = 1.26; 95%: 1.15–1.38)^15^.

In order to understand the relationship between melanoma and prostate cancer, we set out to better describe the relationship between these two common cancers in Australia which is a region of high incidence for both diseases. The current study examined the incidence of PC following a diagnosis of CM, compared to that in the general population, in men from New South Wales, and described factors associated with prostate cancer diagnosis in the group of men who were previously diagnosed with melanoma.

## Results

**Figure 1:**
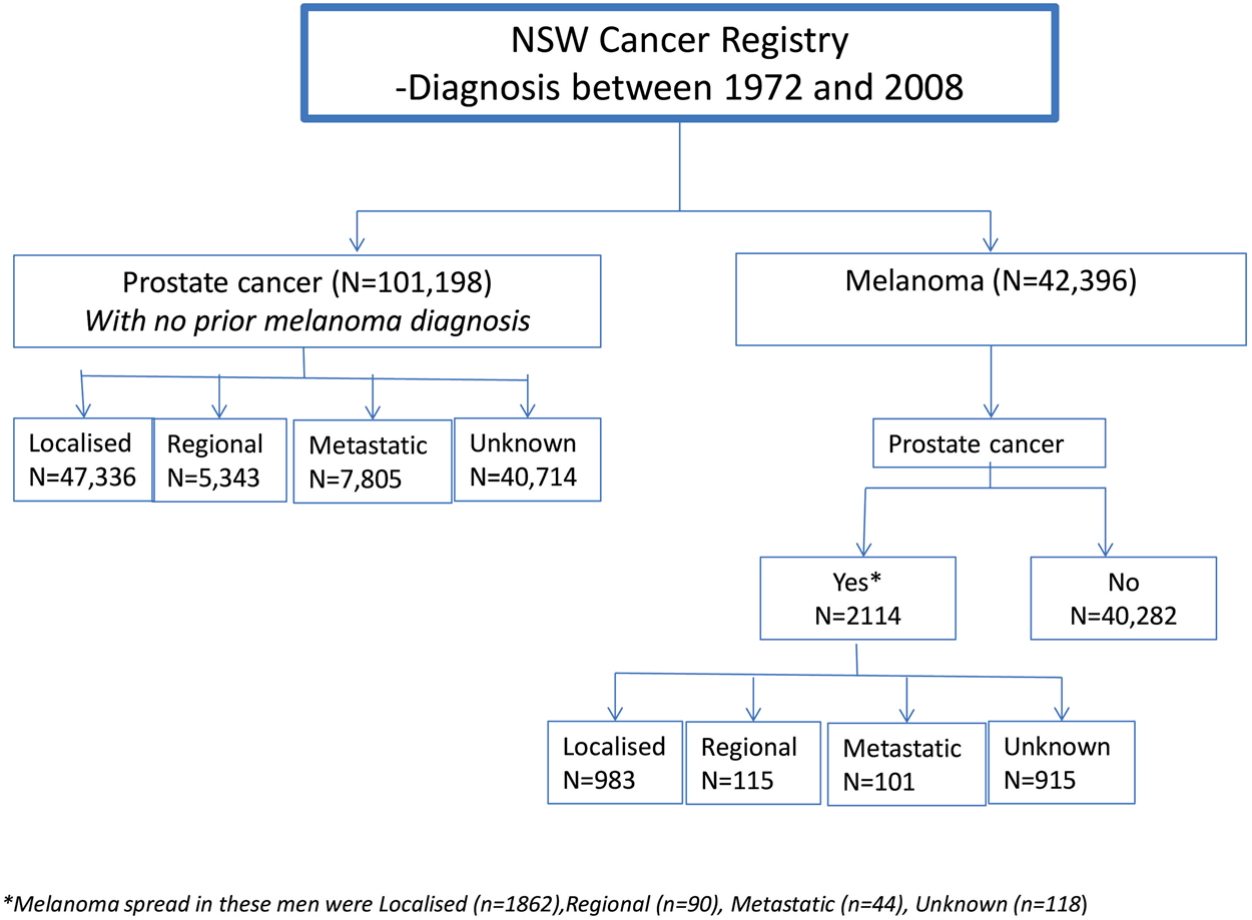
Flow diagram showing the breakdown by disease spread for all melanoma and prostate cancer cases registered in the New South Wales Cancer Registry between 1972 and 2008.

There was a total of 143,594 men diagnosed with melanoma (n = 42,396) or prostate cancer (n = 101,198), between January 1972 and December 2008. Of the men first diagnosed with melanoma, 2114 men were subsequently diagnosed with prostate cancer, as illustrated in Fig. 1. The risk of prostate cancer hereafter, refers to risk in men with a prior diagnosis of melanoma. These cancer cases were categorised by their disease spread for prostate cancer (localised (n = 983), regional (n = 115), metastatic (n = 101), unknown (n = 915)) and melanoma (localised (n = 1,862), regional (n = 90), metastatic (n = 44), unknown (n = 118)).

There was an overall 25% increased risk of prostate cancer in men with a prior diagnosis of melanoma (SIR = 1.25; 95% CI: 1.20–1.31-Table 1) compared to men from the general population, with risk of prostate cancer increased in men diagnosed with localised melanoma (SIR = 1.26; 95% CI: 1.20–1.32), and not in men diagnosed with non-localised or unknown disease. There was little variation in risk observed with increasing Breslow thickness of melanoma (SIR > 1 in all categories), with risk of PC incidence highest in those with Breslow thickness between 0.01–1.00 mm. The overall risk of prostate cancer was increased in all age groups, with risk highest in men aged 60 years or below (SIR = 1.51; 95% CI: 1.30–1.74) and lowest in men aged 80 years and over (SIR = 1.14; 95% CI: 1.04–1.25). There was no significant risk in the diagnosis of prostate cancer in the period between 1972 and 1979, however PC risks were significantly increased in men diagnosed in each of the three periods thereafter. The increased risk of prostate cancer was highest for men who had both cancers diagnosed in the same year (SIR = 14.55 95% CI: 12.59–16.73), and this risk sharply declined thereafter. Notably, there was a significant reduced PC risk for those diagnosed more than 10 years after the melanoma diagnosis. We found little variation in PC risk based on socioeconomic status (SEIFA) and by place of residence, while the risk of PC following melanoma was higher in Australian born men (SIR = 1.62 95% CI: 1.54–1.70), and lower in men born overseas (SIR = 0.54 95% CI: 0.48–0.61).

**Table 1:** Age standardised incidence rates (and 95% CI) for factors associated with prostate cancer risk in men previously diagnosed with cutaneous melanoma, compared to prostate cancer cases without a cutaneous melanoma, for cases diagnosed between 1972 and 2008 in the New South Wales population.

	Expected^a^	Observed^a^	SIR	95%CI	
ALL participants	1687.82	2114	1.25	1.20	1.31
**Spread of disease (melanoma)**					
Localised	1489.33	1878	1.26	1.20	1.32
Regional	64.72	78	1.21	0.95	1.50
Distant	37.02	42	1.13	0.82	1.53
Unknown	96.75	116	1.20	0.99	1.44
**Breslow thickness of melanoma (mm)** ^b^					
0.01–1.00	689.42	940	1.36	1.28	1.45
1.01–2.00	177.34	223	1.26	1.10	1.43
2.00–4.00	127.23	152	1.19	1.01	1.40
>4.00	60.12	75	1.25	0.98	1.56
Missing/Unknown	633.71	724	1.14	1.06	1.23
**Age (years)** ^c^					
<60	119.61	181	1.51	1.30	1.75
60–64	171.30	213	1.24	1.08	1.42
65–69	286.02	385	1.35	1.21	1.49
70–74	343.84	441	1.28	1.17	1.41
75–79	350.58	420	1.20	1.09	1.32
80+	416.47	474	1.14	1.04	1.25
**Period of diagnosis** ^c^					
1972–1979	9.65	10	1.04	0.50	1.91
1980–1989	90.31	117	1.30	1.07	1.55
1990–1999	534.24	656	1.23	1.14	1.33
2000–2008	1053.61	1331	1.26	1.20	1.33
**Follow-up years after CMM diagnosis**					
<1	13.54	197	14.55	12.59	16.73
1–4	225.23	674	2.99	2.77	3.23
5–9	383.20	541	1.41	1.30	1.54
10–14	358.54	313	0.87	0.78	0.98
15+	707.31	389	0.55	0.50	0.61
**Place of birth**					
Australia	1113.91	1803	1.62	1.54	1.70
Overseas	573.91	311	0.54	0.48	0.61
**Socioeconomic status (quintiles)** ^c^					
Q1 (most disadvantage)	469.88	534	1.14	1.04	1.24
Q2	244.14	361	1.48	1.33	1.64
Q3	360.97	445	1.23	1.12	1.35
Q4	340.81	436	1.28	1.16	1.41
Q5 (least disadvantage)	295.84	338	1.14	1.02	1.27
**Place of residence** ^c^					
Major city	1182.74	1473	1.25	1.18	1.31
Inner regional	419.22	516	1.23	1.13	1.34
Outer regional/remote	97.82	125	1.28	1.06	1.52

^a^Prostate cancer cases after a melanoma diagnosis.

^b^Categories based on the 8th edition AJCC Melanoma Staging System guidelines^39^.

^c^Status when diagnosed with prostate cancer.

Negative binomial analyses were undertaken to examine the effect of sociodemographic characteristics and cancer-related factors on the risk of PC diagnosis, for all melanoma cases, after adjusting for age of melanoma diagnosis, stage of melanoma, socioeconomic status, place of birth, place of residence and follow-up period, as shown in Table 2. The incidence rate ratio (IRR) of PC was highest for those diagnosed with melanoma between ages 65–69 (versus < 60; RR = 1.60 95% CI: 1.33–1.92) and lowest for those diagnosed with melanoma aged 80+ (versus < 60; RR = 0.60 95% CI: 0.48–0.75). Compared to those diagnosed with PC within 1–4 years after their melanoma diagnosis, risk of PC diagnosis was highest for those diagnosed with PC in the same year as their melanoma diagnosis (RR = 5.85 95% CI: 4.81–7.12), but there was a gradual reduction in PC risk for those diagnosed with melanoma 5 years prior or longer. PC risk was reduced for those diagnosed with each of the non-localised (regional, metastatic, unknown) spread of melanoma (versus localised), with increasing thickness of melanoma (versus 0.01–1.00 mm) and for those born overseas (versus Australia; RR = 0.30 95% CI: 0.26–0.35). Socioeconomic status and place of residence showed no appreciable difference with PC risk (p-value > 0.05).

**Table 2:** Incidence rate ratio (IRR) and the 95% CI, for factors associated with prostate cancer risk for all cutaneous melanoma cases diagnosed between 1972 and 2008 in the New South Wales population.

	Melanoma (n = 42,398)		Multivariate^b^			
	Prostate cancer (n = 2114)	Prostate cancer-free (n = 40,282)	IRR	95% CI		p-value
**Age groups** ^a^ **-years**						
<60	703	19801	1.00			<0.0001
60–64	332	4410	1.55	1.31	1.83	
65–69	385	4584	1.66	1.41	1.95	
70–74	312	4288	1.31	1.10	1.55	
75–79	205	3607	0.89	0.74	1.08	
80+	177	3592	0.62	0.51	0.76	
**Years of follow up**						
0	197	4372	5.99	5.00	7.17	<0.0001
1–4	674	12704	1.00			
5–9	541	9151	0.40	0.35	0.46	
10–14	313	5659	0.23	0.19	0.27	
15+	389	8396	0.08	0.07	0.10	
**Stage of melanoma diagnosis**						
Localised	1878	33055	1.00			<0.0001
Regional	78	2438	0.55	0.43	0.71	
Distant	42	2408	0.28	0.20	0.40	
Unknown	116	2381	0.69	0.56	0.86	
**Breslow thickness of melanoma (mm)**						
0.01–1.00	940	17727	1.00			0.0016
1.01–2.00	223	4702	0.82	0.69	0.98	
2.01–4.00	152	3348	0.80	0.66	0.98	
>4.00	75	2078	0.75	0.58	0.98	
Missing/Unknown	724	12427	1.08	0.93	1.25	
**Country of birth**						
Australia	1803	25605	1.00			<0.0001
Other	311	14677	0.31	0.27	0.35	
**Place of residence** ^a^						
Major cities	1510	27916	1.00			0.33
Inner regional	478	9657	0.92	0.80	1.05	
Outer regional\Remote\Very remote	126	2709	0.88	0.71	1.10	
**Socioeconomic status** ^a^						
Q1 (most disadvantage)	559	9500	1.00			0.09
Q2	357	6560	0.92	0.77	1.10	
Q3	428	9066	0.82	0.69	0.97	
Q4	420	7824	0.96	0.81	1.15	
Q5 (least disadvantage)	350	7332	0.84	0.70	1.01	

^a^Status when diagnosed with cutaneous melanoma.

^b^Regression analysis adjusted for age of melanoma diagnosis, follow-up years, stage of melanoma, country of birth, place of residence and socioeconomic status.

## Discussion

Our study showed that men in Australia who were previously diagnosed with cutaneous melanoma (CM) had a 25% increased risk of subsequent prostate cancer (PC) diagnosis, compared to men without a previous diagnosis of CM. These results are consistent both in direction and magnitude with previous international findings, reporting an approximate 26% increased risk of PC^14–16^ Of novelty are our findings that PC risk was significantly increased in men with localised CM spread of disease, but not in men with non-localised disease, and that incidence of PC decreased with increasing thickness of melanoma. Our study also showed that PC risk was highest in Australian born men, and in men’s first year of CM diagnosis. These results raise the potential for CM diagnosis to be a predictor of subsequent PC risk.

The increased PC risk following a CM diagnosis and additionally in men who were previously diagnosed with localised CM, but not in non-localised or unknown CM, may be attributed to patterns of care after a CM diagnosis. Early diagnosis and intervention for melanoma is associated with relatively high disease specific survival, and therefore those treated successfully for localised CM may be expected return for regular if not more diligent health screening and monitoring following treatment. Men with a previous diagnosis of CM are more likely to have more regular interactions with primary health care providers and therefore likely to be more vigilant about their health, and consequently this may increase the likelihood of detecting other diseases including PC. Between 2012–2013, the National Health Performance Authority reported more frequent general practitioner (GP) visits in Australia, with increased visits also observed with increasing patients age and having multiple health conditions^17^. This is consistent with our recent observation in a population wide prospective study in NSW, where the likelihood of PSA testing increased with increased frequency of GP visits, and also for those with at least one non-prostate related medical condition (Nair-Shalliker *et al*. *under review*). The reduced risk for those with regional or metastatic CM spread or with increasing Breslow thickness of melanoma is potentially likely due to a lower likelihood of offering prostate cancer testing and/or higher competing causes of death in this group. The reduced risk for those with regional or metastatic CM spread or with increasing Breslow thickness of melanoma, is potentially likely due to a lower likelihood of offering prostate cancer testing, as current guidelines do not recommend screening for prostate cancer in men with a life expectancy of less than 15 years^18–20^.

Incidence of PC was greatest in those diagnosed with PC in the same year as their CM diagnosis, with a subsequent rapid decline in risk after 5 years. This may also be associated with the patterns of care following the initial CM diagnosis. Australian guidelines for skin cancer diagnosis recommend follow-up of melanoma patients should be conducted 3 to 6-monthly for 5 years following the diagnosis of a stage I-III melanoma^13,21^. This increased vigilance in the first 5 years would involve health checks possibly including prostate specific antigen testing (PSA) and digital rectal examination for middle aged or older men, which will increase the likelihood for detecting asymptomatic PC, especially in the first year for all latent or undetected cancers. The reduced risk of PC after 5 years may reflect the decline in surveillance for CM after 5 years or the fact that any men with latent disease may have already been detected shortly after diagnosis.

The higher PC risk in Australian born men compared to those born overseas may be attributed to higher screening rates, including that for PSA testing, in Australian born men compared to men born overseas^22^. Alternatively, it may also be attributed to the variation in CM incidence by birth regions, as CM incidence in NSW is consistently shown to be highest in Australian born men compared to men born in any other regions of the world^23^.

The incidence of PC increased dramatically in Australia when PSA testing, which is publicly funded by Medicare, the universal healthcare system, became available from 1989^24^. In many developed countries a pattern of a greater number of negative biopsies and higher incidence of low to intermediate grade prostate cancer in males aged 50 years and over was documented^25–29^. Thus the increased incidence of PC observed here may be related to the general pattern of PC diagnosis of more low risk disease in a younger population than had been observed prior to widespread PSA testing. However, as the study cohort included all NSW cancer cases diagnosed between 1972 and 2008, it covers a period before the introduction of PSA testing in 1988. Thus, the lack of variation in the risk of PC between the pre- and post- PSA testing period; suggests that the impact of PSA testing in our analysis, although present may be minimal.

A major strength of this study is its representativeness of a population residing in a region of high ambient UV, at time of diagnosis and with a high incidence of prostate cancer. In Australia pathology notification to the Cancer Registry is mandatory, and hence this cohort is fully representative of the NSW population and is the largest cohort of its type that provides high quality incidence data, excellent follow up and additional information on patient and disease related characteristics. Furthermore, the NSWCR is the only Australian registry that systematically collects information on spread of disease at diagnosis and Breslow thickness since 1988. There were a significant proportion of missing data (~34% missing) in the current analysis, which corresponded predominantly with melanoma cases that were diagnosed before 1988. Whilst the large cohort ensures a fully representative sample of men are studied and assists with limiting potential selection biases, there are several factors that were unable to be controlled for. Some cases of melanoma diagnosed prior to 1986 may have not have been registered as mandatory pathology notification to the Cancer Registry was only introduced in 1986. This potential under-reporting will bias the result towards the null as men who may previously have had a melanoma but subsequently diagnosed with prostate cancer will not have been counted in the potentially ‘at risk’ population. There were also a significant number of men who had prostate cancer classified with unknown spread at diagnosis. We examined these cases as a separate group based on recent evidence that suggests that while most of these men will have localised disease, there may be a small proportion with either regional spread and/or metastatic disease, in this group.

The current observation for an increased PC risk in association with CM diagnosis is likely due to patterns of care following a CM diagnosis. These findings also correspond with our previous finding for a positive association between sun exposure and PC risk^9^. Thus, although the reduced risk of PC after 5 years may reflect the drop in surveillance for CM in this, this decline in PC risk after 5 years, may also be attributed to reduced sun exposure due to a change in sun exposure behaviour after a skin cancer diagnosis^9,30^. We have more recently showed a link between melanoma associated pigmentary genes, androgens and serum PSA levels, and proposed a role for androgens in mediating the effect between CM and PC development in populations that have high sun sensitivity^31^. Androgens play a major role in the development of prostate cancer and melanoma, where the relationship between melanoma and prostate cancer may be mediated by testosterone levels^32^. The effectiveness of androgen ablation in the treatment for prostate cancer and potential in treatment for melanoma, confirm the importance of androgens for these diseases^33–35^. Thus the role of androgens in mediating the effect between CM and PC development, while not part of the dataset available within this study should be investigated.

The current findings suggest that CM diagnosis increased the risk of developing subsequent prostate cancer. While part of this increased risk was likely due to greater health surveillance and therefore testing in men with melanoma, additional explanations are possible. This raises the potential for future prostate cancer risk to be discussed with newly diagnosed males with melanoma, but until then further study of potential causes, such as the role of androgens, is needed.

## Materials and Methods

### Study population

This is a retrospective population-wide cohort study set in New South Wales (NSW) Australia, which has an estimated population of ~7.2 million people, making it the most populous state of Australia containing just under one third (32%) of the Australian population^12^. Unit record data for the period 1972–2008 was obtained from the New South Wales Cancer Registry (NSWCR). Notification of cancer diagnosis to the registry is a statutory requirement in NSW. The NSWCR maintains a record of all cases of cancer diagnosed in NSW residents since 1972^36^. The NSWCR has high standards of data completeness and quality, and the data are accepted by the International Agency for Research on Cancer for publication in Cancer Incidence in Five Continents^37,38^. Eligible participants were NSW male residents diagnosed with invasive cutaneous melanoma (ICD-O3 C440-C449) or prostate cancer (ICD-C61), between January 1972 and December 2008.

This study was approved by the NSW Population and Health Services Research Ethics Committee (AU RED Reference number: HREC/13/CIPHS/34). All analyses were performed in accordance with relevant guidelines and regulations using de-identified unit record data files. Individuals’ informed consent was not obtained as a de-identified dataset used in the analysis. Mandatory notification of cancer by pathology laboratories, hospitals, radiation therapy and medical oncology departments, aged care facilities and the Registry of Births, Deaths and Marriages is required under the Public Health Act 2010 and therefore consent was not obtained.

### Data analysis

Data items obtained from the NSWCR were categorised as follows: age at diagnosis of CM (<60, 60–64, 65–69, 70–74,75–79, 80+) years; year of PC diagnosis (1972–1979, 1980–1989, 1990–1999, 2000–2008); follow-up period since melanoma diagnosis (0, 1–4, 5–9, 10–14, 15+) years; spread of disease at presentation (localised, regional, distant metastasis and unknown); country of birth (Australia, Overseas); Breslow thickness was categorised according to the 7th edition AJCC Melanoma Staging System guidelines (0.01–1.00 mm, 1.01–2.00 mm, 2.01–4.00 mm, >4.00 mm, missing/unknown)^39^. Local Government Area (LGA) of residence at time of first primary cancer diagnosis was used to categorize each man into one of five socio-economic status groups according to the Socio-Economic Indexes for Areas (SEFIA-categorised into quintiles) and by remoteness or accessibility using the Accessibility/Remoteness Index of Australia (-major city, inner regional, outer regional/remote)^40^.

### Statistical analysis

We calculated the standardized incidence ratio (SIR) and 95% confidence intervals (95% CI) for (i) the overall incidence rate of prostate cancer diagnosis, and (ii) the incidence rate of diagnosis of prostate cancer by spread of CM, in men with a previous diagnosis of cutaneous melanoma, compared to rates in men without a previous diagnosis of cutaneous melanoma. Expected numbers of new cases of prostate cancer were calculated by predefined age groups and year based on incidence rates for the total population. Person years of risk for a subsequent diagnosis of prostate cancer were accumulated for each subject, ending with either a date of diagnosis of prostate cancer, or the end of the study period (December 2008), whichever came first. Person years at risk were classified for males in five-year age groups and time since entry into the cohort. The expected numbers of prostate cancers were obtained by assuming the background rates observed in the corresponding male general population and applying sex-, age- and calendar specific rates to the appropriate person years at risk. Likewise, the expected numbers of cancers, by disease spread, were obtained by assuming the background rates observed in the corresponding male general population and applying sex-, age- and calendar specific rates to the appropriate person years at risk.

We also examined the association between the socio-demographic and disease related characteristics, and prostate cancer risk within all melanoma cases, using negative binomial regression analysis. In all men previously diagnosed with cutaneous melanoma, we determined the rate ratio (RR) and 95% confidence intervals (95% CI) of developing prostate cancer according to specified socio-demographic categories and disease related characteristics compared to men free of prostate cancer. Patients with unknown area of residence information, and therefore unknown socio-economic status or accessibility/remoteness status, were excluded from this analysis.

Analysis was conducted using SAS (version 9.3, SAS Institute Inc., Cary NC, USA).

### Ethics approval

This study was approved by the NSW Population and Health Services Research Ethics Committee (AU RED Reference number: HREC/13/CIPHS/34).
